# CROSS-TISSUE COMPARISON OF AGING BIOLOGY MEASURES AS NOVEL BIOMARKERS FOR HEALTHY AGING

**DOI:** 10.1093/geroni/igad104.1479

**Published:** 2023-12-21

**Authors:** Reem Waziry

**Affiliations:** Columbia University, New York City, New York, United States

## Abstract

We compared the role of aging biology biomarkers measured in saliva (N=5,808) and blood (N=9,934) as novel biomarkers for prevention of stroke and heart disease in a sample of older adults (aged 50 and older) from the Health and Retirement Study (HRS). Individuals who consented for blood and saliva draw in the HRS 2016 Venous blood and 2008 Telomere length studies were included. Measures in saliva included telomere length. Measures in blood included DNA methylation and physiology biomarkers. The main outcomes included stroke, heart disease and disease-free aging. Cases and disease-free adults were matched on age and sex using a probit link and propensity scores. No association was observed between saliva-based telomere length with stroke or heart disease. In contrast, blood-based biomarkers of aging biology showed strong associations with stroke and heart disease. Biologically older individuals had 3- and 2.5-times higher odds of stroke based on DNA methylation Grim Age (aOR=3.00, 1.87, 4.82, P< 0.001) and Physiology-based Phenotypic Age (aOR=2.50, 1.57, 3.98, P<0.001). Individuals who were biologically older had 3.6- and 2.3-times higher odds of heart disease according to physiology-based Phenotypic Age (aOR=3.64, 95% CI 2.68, 4.94, p < 0.001) and DNA methylation Grim Age (aOR=2.36, 95% CI 1.73, 3.23, P<0.001). Findings of the present study suggest that saliva-based telomere length and blood-based DNA methylation and physiology biomarkers likely capture different aspects of individual aging and vary in their precision and sensitivity as novel biomarkers for prevention of stroke and heart disease in older adults.

